# MRI as a Aiagnostic Tool for Paroxysmal Nocturnal Hemoglobinuria: A Case Report

**DOI:** 10.14744/SEMB.2021.03443

**Published:** 2021-12-29

**Authors:** Bade Von Bodelschwingh, Huseyin Ozkurt

**Affiliations:** Department of Radiology, University of Health Sciences Turkey, Sisli Hamidiye Etfal Training and Research Hospital, İstanbul, Turkey

**Keywords:** Hemochromatosis, hemosiderosis, magnetic resonance imaging, paroxysmal nocturnal hemoglobinuria, radiology

## Abstract

Paroxysmal nocturnal hemoglobinuria (PNH) is a type of hematopoietic stem cell disease and the clinical manifestation of the disease is mainly a combination of anemia and thrombosis. Intravascular hemolysis in PNH leads to hemosiderosis in renal cortex as a rare pattern of hemochromatosis. With this case presentation, we aim to show the radiological findings of this disease.

Paroxysmal nocturnal hemoglobinuria (PNH) is a rare clonal hematopoietic stem cell disease resulting from a somatic gene mutation.^[1]^ The primary manifestations of the disease are hemolytic anemia and thrombosis.^[1,2]^ As a result of intravascular hemolysis, iron accumulates in the cortices of kidneys.^[3]^ From radiological perspective, deposition of iron in the kidneys manifests as signal loss in the renal cortex on T1- and T2-weighted sequences on magnetic resonance imaging (MRI).^[4]^ In this case presentation, we aim to show the radiological findings of PNH disease as it is highly suggestive for the diagnosis as well as the importance of implying the iron deposition in specific organs on MRI, although being incidental, allows the clinician to put the treatment on the right track.^[5]^ This case report was published with consent of the patient.

## Case Report

A 64-year-old man was admitted to the emergency service with complaint of the right upper quadrant pain, jaundice, and darkening of the urine for 3 days. He had hypertension and a history of coronary by-pass surgery. He had no history of drug use.

On physical examination, the right upper quadrant tenderness and newly-emerging periumbilical ecchymosis were observed. The Murphy sign was negative. On laboratory examination, hepatic function tests were moderately high (Aspartate Transaminase: 348 and Alanine Aminotransferase: 231). Very high lactate dehydrogenase (LDH) levels and increased C-reactive protein (CRP) were observed (59 mg/L). He had minimal direct bilirubinemia (total: 1 mg/dL direct.0.29 mg/dL). Urine test showed 3+ protein and 2+ ketone. According to the clinical findings, the initial diagnosis was suspected the cause in the biliary system. Ultrasound examination revealed biliary stones and mild diffuse wall thickness measuring 4 mm. There was no fluid around the gallbladder. No evidence of ductal dilatation.

**Figure 1.**
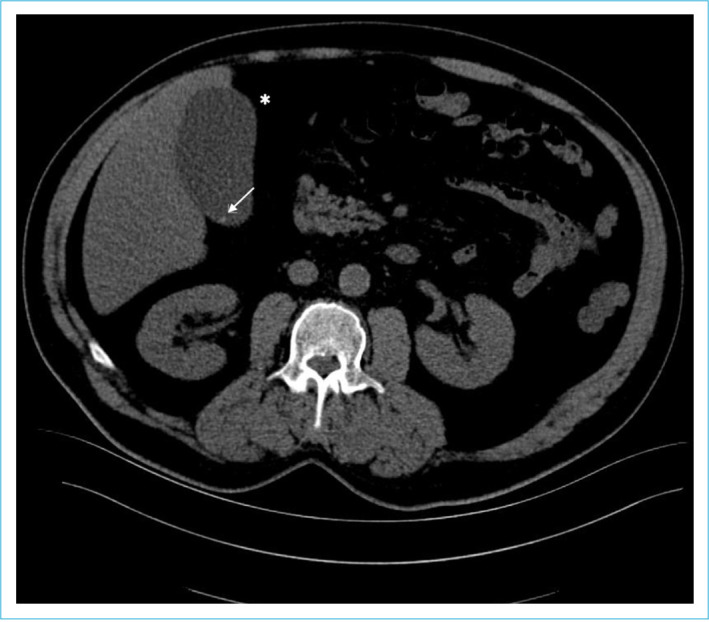
Axial non-contrast CT image shows no abnormal finding related to iron accumulation in kidneys. Note hydropic gallbladder (star) and millimetric gallstone (arrow).

On computed tomography (CT) scan without contrast material, gallbladder was distended and stones, which were millimetric in size, was seen (Fig. 1). There was no other relevant finding. The patient was hospitalized with the differential diagnosis of cholecystitis. In 3 days hemoglobin levels went down from 13.9 to 8.3 g/dL. There was a significant increase in creatine, LDH, CRP, and total bilirubin levels. These findings were consistent with hemolytic anemia. Coombs test were negative.

To exclude biliary pathologies, abdomen MRI and magnetic resonance cholangiopancreatography were planned. The images were obtained with a commercial 1.5 Tesla MRI machine (Avanto, Siemens, Germany) using axial T1-weighted spin echo, axial in-phase and out-of-phase T1-weighted gradient-echo and axial T2-weighted spin echo with and without fat suppression (BLADE), coronal T2-weighted spin echo (HASTE), coronal T2-weighted gradient echo (TRUFI), and single slab 3D turbo spin echo (SPACE) sequences.

On MRI images, there was no evidence of acute biliary pathologies but the bilateral signal of the renal cortex was markedly and diffusely low on T2-weighted images comparing to the signal of medulla consistent with hemosiderin accumulation. On in-phase sequence, prominent signal loss in renal cortex was also noted comparing to out-of-phase images. In the light of these MRI findings and clinical history, we recommended further investigation for PNH disease. The flow cytometry analysis of peripheral blood showed deficiency of glycosylphosphatidylinositol-anchored proteins (GPI-APs). The bone marrow biopsy revealed only reticulocytosis. These findings were consistent with classical PNH. The patient has been followed with eculizumab therapy since then.

## Discussion

PNH is a type of hemolytic anemia resulting from a clonal expansion of hematopoietic stem cells that have a somatic mutation of the X-linked gene phosphatidylinositol glycan-class A. This mutation leads to deficiency of GPI-APs on the progeny of mutated stem cells’ cell membrane. Two of these proteins named CD55 and CD59 normally serve as complement regulatory proteins.^[1,2,6-8]^ Therefore reduction or absence of these proteins makes red cells more prone to complement-mediated lysis which causes the main manifestation of this disease, hemolytic anemia.^[2]^ The absence of CD55 causes extravascular hemolysis, whereas absence of CD59 causes intravascular hemolysis, which is more prominent in the course of the disease.^[1]^ This presents itself usually as anemia, increased reticulocytes, and LDH levels.^[9]^ The less common but the main reason of mortality is thrombosis. Intra-abdominal venous thrombosis such as Budd-Chiari syndrome occurs more often than thrombosis at the other cites.^[9]^ Other manifestations of the disease are dysphagia, dyspnea, abdominal cramps and chronic renal failure.^[6,9]^ Major diagnostic tool for the suspected disease is flow cytometry to show the granulocyte PNH clones.^[10]^ The first line of treatment is eculizumab with the aim of reduction of intravascular hemolysis and thrombosis.^[6]^

**Figure 2.**
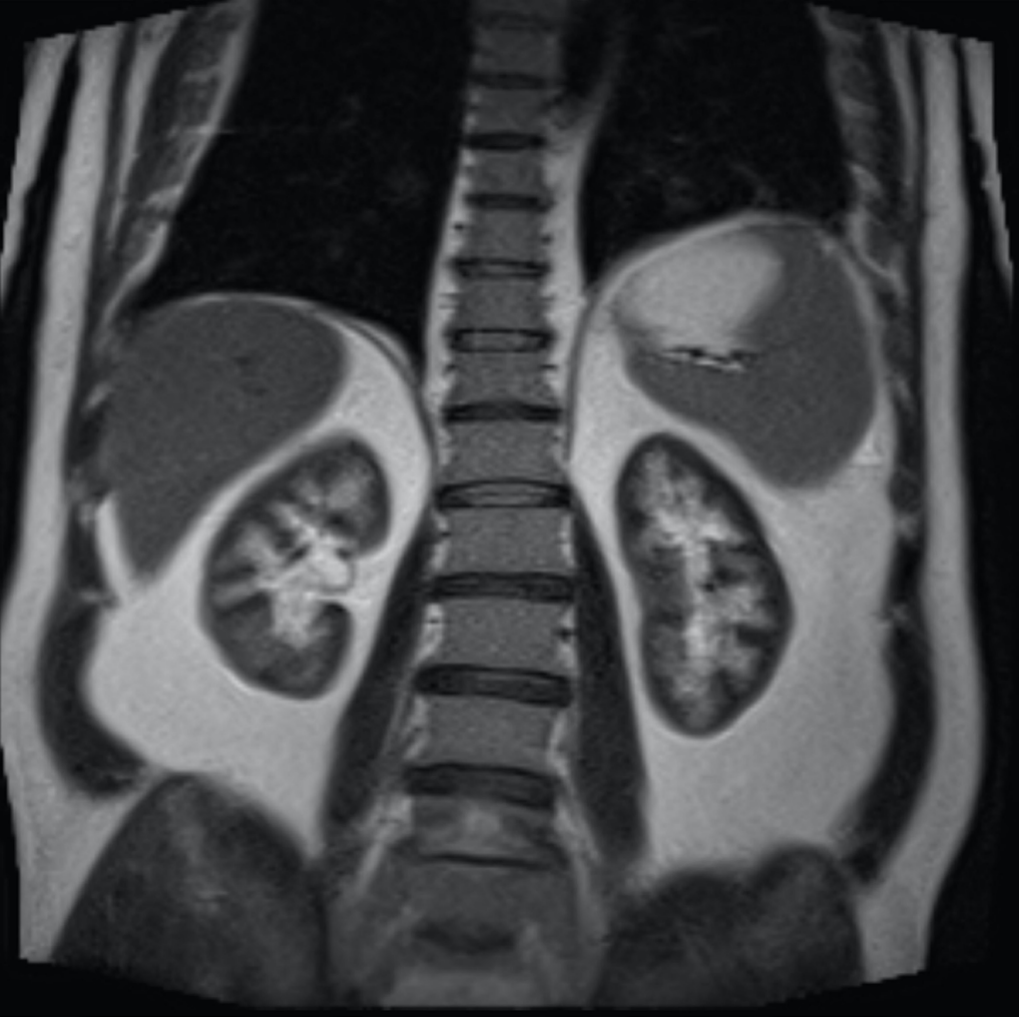
On MRI coronal T2-weighted images show prominent signal decrease of cortices of both kidneys and reversal of signal of cortex and medulla.

**Figure 3.**
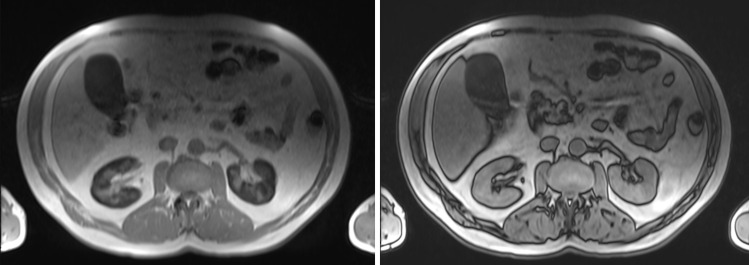
On MRI signal decrease is shown in the cortices of the kidneys on in-phase image comparing **(a)** to out-of-phase image **(b)**.

Iron metabolism in human body is very a sophisticated system controlled by several different genes and proteins.^[11]^ There is no excretion mechanism for this element other than desquamation of epithelial cells and bleeding.^[12,13]^ Iron regulation can be affected by many diseases resulting with accumulation or deficiency.^[13]^ Accumulation of this element in organs classified as primary and secondary hemochromatosis can be observed due to various diseases.^[5]^ The nature of the disease also determines the pattern of iron accumulation. Renal iron deposition is a rare and highly specific type of these patterns.^[5]^ PNH causes hemosiderin deposition in proximal convoluted tubules of the kidneys.^[3]^ In all radiological examinations, MRI is the most sensitive imaging method to show the iron deposition in organs. Because of the supermagnetic properties of ions, T1 and T2 relaxation time shortens and can be evaluated as a loss of signal intensity.^[5]^ Normally in T1-weighted pulse MRI sequences, the renal cortex-medulla differentiation can be depicted easily as cortex signal higher than the medulla. In PNH, accumulation of hemosiderin in renal cortex causes the signal decrease resulted as cortical hypointensity comparing to medulla. As for the T2 weighted sequences, normal cortex and medulla show high-signal intensity making the differentiation difficult.^[4,7]^ Again the iron accumulation lowers the cortical T2 signal intensity, whereas medulla signal remains the same.^[4]^ In our case, these signal properties of kidneys were highly prominent suggesting the diagnosis (Fig. 2). Diagnosis of iron overload can also be done by in-phase/out-of-phase sequences. Tissues, in which iron accumulates show signal loss on in-phase sequences comparing to the out-of-phase sequence, while normal tissues do not show any significant signal loss.^[5]^ This finding was depicted in our case (Fig. 3).

In PNH patients there is no iron deposition in other cites such as liver or spleen,^[5]^ unless the patient receives blood transfusion or has a history of hepatic or portal venous thrombosis.^[9]^ Our patient had been given blood transfusion for the first time at the period of hospitalization. As a result on MRI, there was no sign of iron deposition in other organs. In our case sonographic findings are suspicious for cholecystitis making the diagnosis challenging. Although in PNH, there is no specific sonographic finding; US can be useful for patients developing complications such as deep venous thrombosis, Budd-Chiari syndrome and biliary stones. On non-contrast CT, high attenuated renal parenchyma was previously described.^[4]^ This finding was absent in our case (Fig. 1).

## Conclusion

The diagnosis of PNH can be challenging due to nonspecific symptoms and clinical manifestations of the disease. MRI can be used effectively to shorten the list of differential diagnosis, as the dark signal of renal cortex on T1- and T2-weighted images is highly characteristic. After diagnosis of PNH is made, other imaging modalities can be used for suspected complications.

### Disclosures

**Informed consent:** Written informed consent was obtained from the patient for the publication of the case report and the accompanying images.

**Peer-review:** Externally peer-reviewed.

**Conflict of Interest:** None declared.

**Authorship Contributions:** Concept – B.V.B., H.O.; Design – B.V.B.; Supervision – H.O.; Materials – H.O.; Data collection &/or processing – B.V.B.; Analysis and/or interpretation – B.V.B.; Literature search – B.V.B., H.O.; Writing – B.V.B.; Critical review – H.O.
